# Exploring the impact of various zwitterionic surface modifications on the mucus diffusion and membrane permeability of lipid-based nanocarriers

**DOI:** 10.1007/s13346-025-01990-x

**Published:** 2025-10-09

**Authors:** Antonio Spennacchio, Luca Maurice Richter, Daniel Stengel, Martin Hermann, Angela Assunta Lopedota, Antonio Lopalco, Nunzio Denora, Andreas Bernkop-Schnürch

**Affiliations:** 1 Department of Pharmaceutical Technology, Institute of Pharmacy, Center for Chemistry and Biomedicine, University of Innsbruck, Innsbruck, Austria; 2 Department of Pharmacy - Pharmaceutical Sciences, University of Bari “Aldo Moro”, Bari, Italy; 3 Department of Anesthesia and Intensive Care, Medical University of Innsbruck, Innsbruck, Austria

**Keywords:** Zwitterionic surfactants, Nanoemulsions, Mucus diffusion, Cellular uptake, Toxicity assessment, Flow cytometry

## Abstract

This study investigates the effect of structural differences among six zwitterionic surfactants on the performance of lipid-based nanoemulsions. Nanoemulsions were prepared using a standardized formulation approach to minimize compositional variability, thereby isolating the influence of surfactant type. The headgroups of the zwitterionic surfactants, phosphorylcholine, carnitine, amine oxide, betaine, and sulfobetaine, were evaluated for their impact on key parameters including physical stability, cytotoxicity, mucus diffusion, and cellular uptake within the overall surfactant structure. Results revealed significant variations in nanoemulsion performance depending on the zwitterionic surfactant structure. Formulations containing the surfactant cocamidopropyl hydroxysultaine exhibited moderate cytotoxicity but demonstrated superior diffusion through porcine mucus and enhanced uptake by human cells. In contrast, n-dodecylphosphocholine-based nanoemulsions showed excellent stability across all tested biorelevant media coupled with low cytotoxicity. Lipid-based nanocarriers formulated with lauryldimethylamine oxide combined good stability in biorelevant media with high mucus diffusion and moderate cellular uptake. Lauryl betaine-based systems displayed low hemolytic and moderate cytotoxic activities, but showed limited mucus penetration and cellular uptake. This study elucidates critical differences among various zwitterionic surfactants and highlights their suitability for liquid pharmaceutical formulations in the nanoscale range.

**Supplementary Information:**

The online version contains supplementary material available at 10.1007/s13346-025-01990-x.

## Highlights

Zwitterionic surfactants with distinct headgroup architectures were systematically evaluated for their impact on lipid-based nanoemulsions.

Variations in chemical groups and charge positioning within zwitterionic surfactant structures significantly influenced stability, toxicity, and mucus permeability.

**Supplementary Information:**

The online version contains supplementary material available at 10.1007/s13346-025-01990-x.

## Graphical abstract

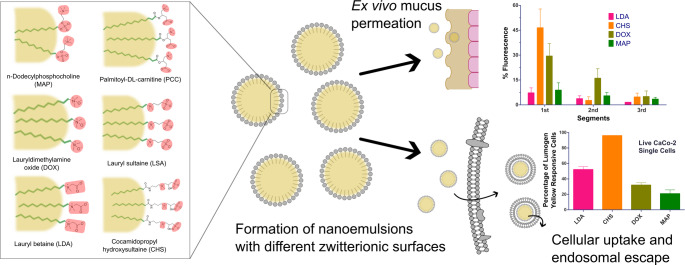

## Introduction

Drug delivery via mucosal membranes occupies a position of considerable importance in the field of delivery science. In addition to the convenience and simplicity of application, the broad availability and accessibility to patients worldwide further underscore the need to direct future research toward innovative delivery systems targeting mucosal tissues. Despite recent advances, mucosal administration remains a formidable challenge due to the presence of mucus and absorption barriers. The dense, viscoelastic network of mucins can trap drug particles, thereby reducing their permeability and ultimately limiting their bioavailability [1, 2]. Additionally, mucosal membranes are generally located within cavities that present harsh conditions for drug stability, often compromising the integrity of the delivery system prior to absorption [3, 4].

The zwitterionic nature of most cellular membranes and viral surfaces has inspired the development of zwitterionic coatings for nanocarriers to impart bioinert properties. These coatings enhance diffusion through mucus and tissues, exhibit strong anti-fouling behavior, and reduce opsonization. Their bioinert characteristics stem from the strong ionic structuring of water molecules around the zwitterionic groups, resulting in highly hydrophilic surfaces. In addition, zwitterionic coatings maintain an internally balanced surface charge, typically yielding a near-neutral zeta potential. This charge balance minimizes the formation of ion pairs with charged components of mucus, the extracellular matrix, cell membranes, or blood, as no free ions are released from the surface [5].

Unlike PEGylated surfaces, zwitterionic materials can bind up to eight times more water molecules per repeating unit and remain stable without aggregation, even under high-salt conditions [6]. Moreover, they do not significantly increase the hydrodynamic diameter of nanocarriers and do not generate peroxides or reactive oxygen species, issues commonly associated with PEG [7]. Notably, zwitterionic nanocarriers have demonstrated higher cellular uptake and membrane permeation compared to PEGylated counterparts [8].

The ability of certain viruses, such as poliovirus, Norwalk virus, and human papillomavirus, to traverse the mucus barrier without hindrance has further motivated the adoption of zwitterionic surface designs for nanocarriers. Several studies have shown that such modifications can improve diffusion through the mucus gel layer and enhance in vivo performance following mucosal administration [9–12].

Despite the increasing use of zwitterionic surfaces, there is considerable structural variability among different zwitterions, particularly in the chemical composition of their headgroups and the spatial arrangement of their positive and negative charges. These structural differences are likely to significantly influence the performance of nanocarriers. However, to date, a systematic evaluation of these effects has not been conducted.

**Fig. 1:**
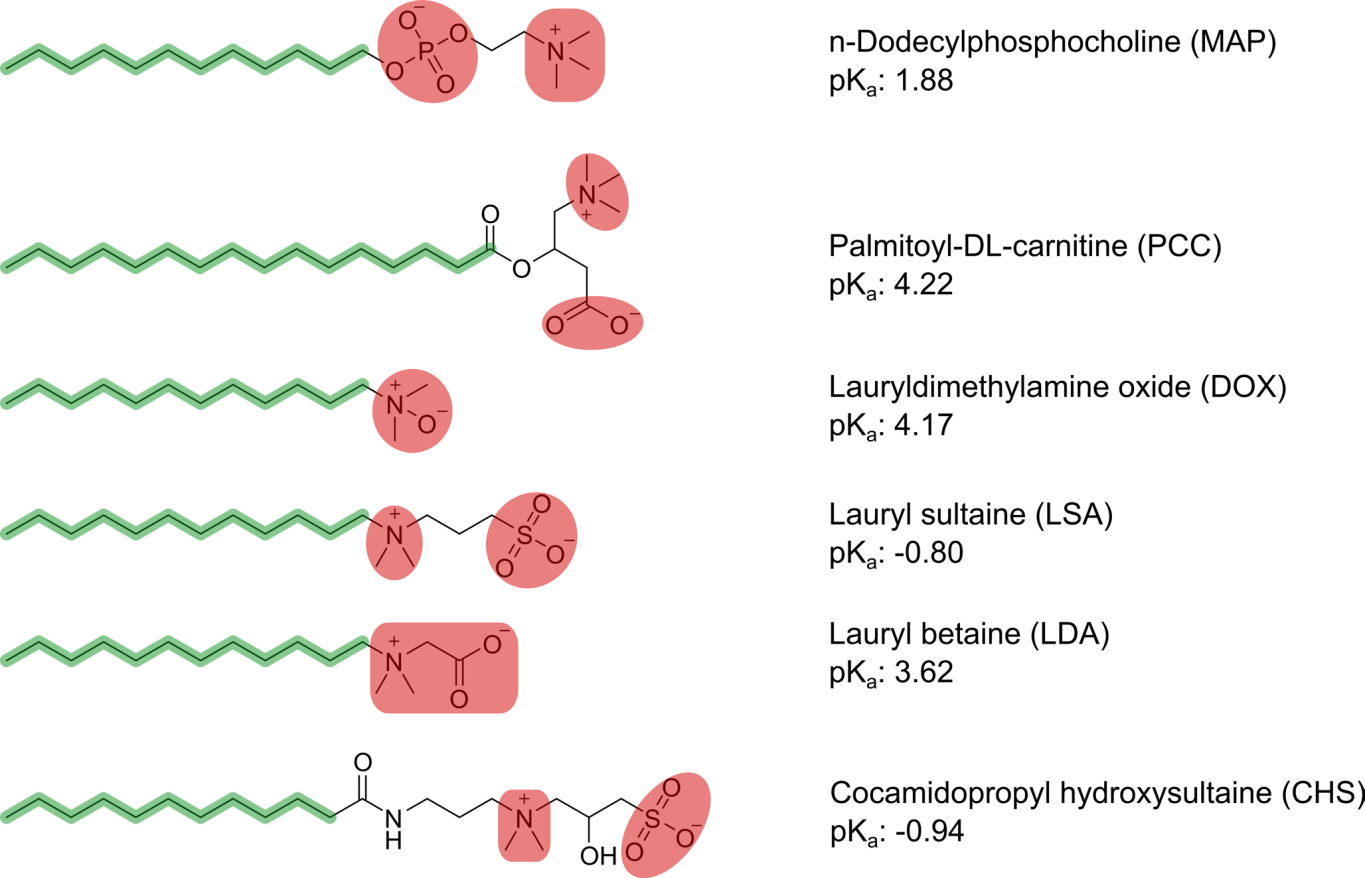
Structural formulas of the selected zwitterionic surfactants; highlighted in green are the hydrophobic moieties of the surfactants, highlighted in red are the zwitterionic structures

This study aims to investigate how structural variations in zwitterionic surfaces influence the performance of nanocarriers for mucosal drug delivery. Among the various nanocarrier systems, lipid-based nanocarriers, such as liposomes, lipid nanoparticles (LNPs), solid lipid nanoparticles (SLNs), nanostructured lipid carriers (NLCs), and lipid nanoemulsions, offer a distinct advantage: they can be readily functionalized with a range of zwitterionic surface modifications by incorporating different zwitterionic surfactants. As shown in Fig. 1, six zwitterionic surfactants were selected, each featuring unique chemical compositions and charge distributions within their zwitterionic headgroups. These surfactants were used to formulate lipid nanoemulsions with droplet sizes ranging from 50 to 250 nm. The study assessed key performance parameters, including physical stability in biorelevant fluids, cytotoxicity, mucus permeation via diffusion studies, and membrane permeation via cellular uptake studies, to determine whether structural differences among zwitterionic surfactants (ZS) translate into functional differences. Additionally, mechanistic studies were performed to better understand the underlying factors driving the observed behaviors, thereby providing critical insights to inform future design of zwitterionic nanocarriers.

## Materials and methods

### Materials

Captex^®^ 355 EP/NF (medium-chain triglycerides, CPT) and Capmul^®^ MCM C8 (mono and diglycerides of medium chain fatty acids, CPM) were kindly donated by Abitec (Columbus, USA). Transcutol^®^ HP (TSC) was provided by Gattefossé SAS (Saint-Priest Cedex, France). Bovine serum albumin (BSA) (≥ 96%), 96% (V/V) ethanol (EtOH), minimum essential medium eagle (MEM), pepsin from gastric mucosa, potassium chloride, resazurin sodium salt and Triton X-100 were supplied by Sigma-Aldrich (Vienna, Austria). 4-(2-Hydroxyethyl)- 1-piperazinethansulfonic acid (HEPES) (≥ 99.5%) was obtained from ROTH GmbH (Karlsruhe, Germany). D-Glucose anhydrous and sodium chloride (≥ 99.5%) were received from VWR chemicals (Solon, USA). Phosphate buffered saline (PBS), penicillin, streptomycin, and fetal bovine serum (FBS) were obtained from Merck (Tutzing, Germany). Lumogen^®^ F Red 305 and Lumogen^®^ F Yellow 083 were a kind gift from BASF (Ludwigshafen, Germany). Cell culture supplies were purchased at Biochrom GmbH (Berlin, Germany). Sodium taurocholate hydrate was purchased from ABCR GmbH (Karlsruhe, Germany) and lecithin (Lipoid S100) was provided by Lipoid GmbH (Ludwigshafen, Germany). n-Dodecylphosphocholine (MAP) was purchased from Avanti Polar Lipids, LLC (Alabaster, AL, USA), cocamidopropyl hydroxysultaine (CHS) and lauryldimethylamine oxide (DOX) were purchased from Biosynth Laboratories Ltd. (Billingham, UK), palmitoyl-DL-carnitine chloride (PCC) was purchased from Cayman Chemical Company (Ann Arbor, MI, USA), lauryl betaine (LDA) was purchased from Sigma-Aldrich (Saint-Louis, MO, USA), lauryl sultaine (LSA) was purchased from Carl Roth GmbH & Co. KG (Karlsruhe, Germany).

### Preparation of preconcentrates

Lipid-based nanocarrier preconcentrates were formulated by combining CPM or a 1:1 mixture of CPM with CPT as the oil phase, with ZS dissolved in a co-solvent (EtOH or TSC). The mixtures were maintained at 50 °C and 1200 rpm using an Eppendorf ThermoMixer F^®^ (Eppendorf, Hamburg, Germany) to achieve a homogeneous phase. Similar to a previously described method, stability was assessed by visually inspecting the final preconcentrates for phase separation and component precipitation following centrifugation at 10,000 rcf for 10 min at 25 °C. Only stable preconcentrates were selected for nanoemulsion (NE) formation [13]. HLB values were calculated for the surfactants based on the system described by Griffin and following equation [14]:

$$HLB = 20 \cdot \frac{{{m_H}}}{m}$$

wherein ${m_H}$ is the mass of all hydrophilic parts of the surfactant, and $m$ is the total mass of the molecule.

### Preparation and characterization of nanoemulsions

Preconcentrates were diluted at a 1:100 ratio in HEPES buffer (HBS) containing 1 g/L glucose (as nutrient), 25 mM HEPES, 5 mM KCl, 136.7 mM NaCl, and 1 mM CaCl_2_ (pH 7.4), and emulsified for 5 min using an ultrasonic processor at 80% amplitude (UP200H, Hielscher Ultrasonics GmbH, Teltow, Germany), or allowed to self-emulsify (in the case of CHS3 and CHS4) to obtain NE. Droplet size, polydispersity index (PDI), and zeta potential were subsequently determined by dynamic and electrophoretic light scattering (Zetasizer Nano ZSP, Malvern Instruments, Worcestershire, UK) at 37 °C. Zeta potential was measured in triplicate using a dip cell equipped with a palladium electrode (Malvern Universal Dip Cell, Worcestershire, UK). Only NE with a droplet size below 250 nm and a PDI below 0.3 were considered acceptable. Zeta potential at pH 1.2 was determined by electrophoretic light scattering at 37 °C after preparing the nanoemulsions at a ratio of 1:100 in diluted hydrochloric acid (pH 1.2) as described above.

### Stability in simulated biorelevant fluids

To ensure the stability of the NE during the experimental period, initial stability assessments were conducted in HBS (pH 7.4). Droplet size and PDI were measured at 0, 4, and 24 h while the formulations were maintained under controlled conditions (37 °C, 350 rpm) in a thermomixer. Additionally, stability studies were performed under simulated in vivo conditions. Preconcentrates were emulsified to achieve a final concentration of 1% (v/v) in fasted state simulated intestinal fluid (FaSSIF, pH 6.5) and fasted state simulated gastric fluid (FaSSGF, pH 1.2), both in the absence and presence of 0.32% (w/v) pepsin. Changes in droplet size and PDI were monitored over a 24-hour period. Furthermore, to evaluate NE interaction with mucus, a 0.1% (w/v) mucus solution was prepared by dispersing porcine mucus in HBS (pH 6.8). The solution was stirred for one hour, sonicated for 10 min, and centrifuged at 10,000 rpm for 10 min (Sigma 3-18KS, Sigma Laborzentrifugen GmbH, Osterode am Harz, Germany). Diluted NE (1.0%, v/v) were then homogenized in equal volumes with the prepared mucus solution, and the samples were incubated at 37 °C and 350 rpm for 4 h. Droplet size and PDI were monitored over a 24-hour period. For studies involving interactions with BSA, a 1% (w/v) BSA solution was homogenized in equal volumes with 1.0% (v/v) NE. The droplet size and PDI of the resulting formulations were analyzed as described above.

### Mucus collection and purification

Freshly excised porcine intestine was obtained from a local slaughterhouse, and mucus was carefully scraped from the intestinal wall. Segments containing food residues or yellow-tinged mucus were excluded. The collected mucus was initially stored at −20 °C prior to purification. For purification, the crude mucus was diluted 1:5 in 0.1 M sodium chloride and stirred for one hour at 10 °C. Following centrifugation at 10,000 rpm and 4 °C for 2 h (Sigma 3-18KS, Sigma Laborzentrifugen GmbH, Osterode am Harz, Germany), the supernatant was discarded. The mucus was then re-diluted with half the original volume of 0.1 M sodium chloride, and the centrifugation step was repeated. The purified mucus was stored at −20 °C until further use.

### Mucus penetration

Mucus penetration depth was assessed using a modified rotating cylinder method, based on a previously established setup [15, 16]. Silicone tubes (5 cm in length, 4 mm inner diameter) were filled with mucus and sealed at one end. Preconcentrates labeled with Lumogen^®^ F Red 305 at a concentration of 1 mg/mL were evaluated to ensure consistent fluorescence intensity after dispersion in HBS (pH 6.8) at a concentration of 1% (v/v). For each NE formulation, 50 µL of the 1% (v/v) dispersion in HBS (pH 6.8) were carefully applied to the open end (sample segment) of the mucus-filled tubes. The tubes were then completely sealed and rotated horizontally at 50 rpm for 24 h at 37 °C.

Following incubation, the tubes were frozen at −80 °C and sectioned into 2 mm segments. The sample segment was mechanically removed to avoid false positive detection of sample which did not penetrate into the mucus. To extract the lipophilic fluorescence marker, each segment was soaked in 350 µL of EtOH, ultrasonicated for 1 h, and subsequently stirred for 24 h under light protection. Samples were then centrifuged at 13,400 rpm for 5 min, and 100 µL of the supernatant were analyzed using a spectrophotometer (Tecan, Salzburg, Austria) with excitation at 570 nm and emission at 640 nm. NE diluted 1:8 with EtOH served as the reference standard for maximum fluorescence, while HBS (pH 6.8) was used as the blank. The blank’s fluorescence intensity was subtracted from sample readings.

### Hemolysis assay

To simulate the interaction of NE with biological membranes and simulate endosomal escape, an in vitro hemolysis assay was conducted [8]. Erythrocytes were diluted 1:200 (v/v) with sterile HBS (pH 7.4). NE, at concentrations ranging from 0.01% to 0.1% (v/v), were added in 200 µL aliquots to 200 µL of the erythrocyte suspension. Samples were then incubated in an orbital shaker (Orbital Shaker-Incubator ES-80, Grant Instruments Ltd, Cambridge) at 37 °C and 150 rpm for 4 and 24 h. Following incubation, the mixtures were centrifuged at 8,000 rpm for 10 min (MiniSpin centrifuge, Eppendorf, Hamburg), and the absorbance of the supernatants was quantified by UV spectroscopy at 415 nm. Triton X-100 at 1% (v/v) and HBS buffer served as positive and negative controls, respectively. Hemolysis was calculated using the following equation (Eq. 1):

1 $$\displaylines{ Hemolysis\left[ \% \right] = \cr \frac{{Abs\left( {sample} \right) - Abs\left( {negative\:control} \right)}}{{Abs\left( {positive\:conrol} \right) - Abs\left( {negative\:control} \right)}} \cr \times 100 \cr} $$

### Cell studies

Caco-2 cells, a widely accepted in vitro model for the intestinal epithelium, were cultivated to form a confluent monolayer in MEM, supplemented with 10% (v/v) heat-inactivated FBS and 1% (v/v) penicillin-streptomycin solution. Cells were maintained at 37 °C in a humidified atmosphere containing 5% CO_2_ until reaching full differentiation, typically after 21 days of culture. The resulting monolayers were used for subsequent experiments involving cytotoxicity assays, as well as studies on cellular interaction and uptake of NE.

#### Resazurin assay

Cytotoxicity of NE was evaluated using the resazurin assay on Caco-2 cells. Cells were seeded at a concentration of 2 × 10⁵ cells/mL in 96-well plates and cultured for three days until confluent. The monolayers were washed twice with sterile HBS (pH 7.4). Subsequently, cells were incubated with 100 µL of NE diluted in HBS at concentrations ranging from 0.010% to 0.100% (v/v). After 4 h of incubation, cells were washed twice with HBS before being treated with 150 µL of a 44 µM resazurin solution for 2 h. Following incubation, 100 µL of the supernatant were transferred to black 96-well plates, and fluorescence intensity (FI) was measured at an excitation wavelength of 540 nm and an emission wavelength of 590 nm using a Tecan Infinite M200 microplate reader (Salzburg, Austria).

HBS served as the positive control, and 0.100% (V/V) Triton X-100 was used as the negative control. Cell viability was calculated according to the following formula (Eq. 2):

2 $$\displaylines{ Cell\:viability\left[ \% \right] = \cr \frac{{FI\left( {sample} \right) - FI\left( {negative\:control} \right)}}{{FI\left( {positive\:control} \right) - FI\left( {negative\:control} \right)}} \cr \times 100 \cr} $$

#### Cellular uptake

The impact of ZS on cellular uptake was evaluated using flow cytometry. Cellular uptake studies were conducted on Caco-2 cells. Cells were seeded at a concentration of 5 × 10⁴ cells/mL in 24-well plates and cultured for ten days until confluent. Preconcentrates were loaded with 0.15% (w/v) Lumogen^®^ F Yellow 083, a lipophilic marker (log *P* > 10). Lumogen^®^ F Yellow 083-labeled preconcentrates were diluted in sterile HBS buffer (pH 7.4), yielding a final concentration of 0.010% (v/v). Cells were incubated with 500 µL of the diluted NE for 4 h at 37 °C. Cells were washed twice with sterile HBS buffer to remove sample and dye remnants. Following incubation, cells were detached using 150 µL of trypsin-EDTA solution (0.05%/0.02% in PBS) and incubated at 37 °C for 5 min. Enzymatic activity was quenched by adding 500 µL of MEM. Suspensions from two wells were combined in a 15 mL Falcon tube after gentle pipetting to disperse the cells. Samples were centrifuged at 800 rpm for 4 min, and the supernatant was discarded. The cell pellet was resuspended in 3 mL of cold PBS (100 mM, pH 6.8). This washing step was repeated twice, after which the final cell pellet was resuspended in 500 µL of PBS (100 mM, pH 6.8). Cell suspensions were filtered through a 70 μm cell strainer and analyzed using a Fortessa LSR II flow cytometer (BD Biosciences, USA). Cells treated with HBS alone served as the blank control. Voltages were adjusted for forward and side scatter (FSC/SSC) and applied uniformly to all analyzed samples. A minimum of 100,000 cells per sample was analyzed. Data were processed using FlowJo™ v10.8 software, and the percentage of Lumogen^®^ F Yellow 083-responsive cells was determined within the population of viable single cells.

#### Cell internalization with fluorescence and confocal microscopy

Cellular interactions of the formulations were investigated via widefield fluorescence and confocal microscopy using Caco-2 cells. Cells were seeded at a concentration of 5 × 10⁴ cells/well in 8-well ibidi slides (ibidi GmbH, Gräfelfing, Germany). Cells were cultured until a confluency of 70–90% was reached. Preconcentrates of the NE were loaded with 0.15% (w/v) Lumogen^®^ F Yellow 083 for optical visualization of the samples. Labeled preconcentrates were diluted in sterile HBS buffer (pH 7.4), yielding a final concentration of 0.010% (v/v). Cells were incubated with 200 µL of the diluted NE for 4 h at 37 °C, and subsequently, washed twice with sterile HBS buffer in order to remove sample remnants and dye. Confocal microscopy samples were stained with a 5 µg/mL Hoechst 33342 solution in sterile HBS for 20 min at 37 °C. Fluorescence images were acquired using an Olympus IX70 inverted fluorescence microscope equipped with a UC90 camera and CellSens Standard software (Olympus, Nagano, Japan). Confocal images were acquired with a Leica TCS SP8 gSTED on the LAS X software (Leica Microsystems GmbH, Wetzlar, Germany). The brightness of the acquired images was digitally adjusted.

### Statistical analyses

Results are expressed as the mean ± Standard Deviation (S.D.) from independent experiments. For the flow cytometric study statistical significance was calculated using a one-way analysis of variance (ANOVA) test followed by Bartlett’s test (GraphPad Prism version 9.3). Differences were considered significant at the *p* < 0.0001 level.

## Results and discussions

### Preparation and selection of nanoemulsions

Six different ZS, MAP, LDA, DOX, CHS, PCC, and LSA, were selected for the formulation of NE (Fig. 1). A systematic screening of oil phases and co-solvents was conducted to achieve two primary objectives: (1) identifying an oil phase compatible with all selected surfactants and co-solvents, and (2) developing preconcentrates with similar compositions to allow head-to-head comparisons of the resulting NE, thereby isolating the effect of surfactant type on formulation properties. CPM, composed of mono- and diglycerides of medium-chain fatty acids (mainly caprylic acid), was used as the oil phase due to its well-documented emulsification and stabilization properties [17, 18]. CPM was selected based on its compatibility with the surfactant CHS, a compatibility that was not observed with other tested oil phases. However, CPM alone proved unsuitable for formulating with MAP, LDA, or DOX. To address this limitation, a 1:1 mixture of CPM and CPT, a triglyceride composed primarily of medium-chain fatty acids, was used for the other ZS. This combination ensured high compatibility for MAP, LDA, and DOX formulations while maintaining compositional similarity across preconcentrates. EtOH and TSC, a highly purified diethylene glycol monoethyl ether, were selected as co-solvents based on their compatibility with the surfactants and their established roles in pharmaceutical formulations [19]. These co-solvents facilitated the formation of homogeneous preconcentrates and contributed to the stabilization of the resulting NE by reducing interfacial tension between the oil and aqueous phases. The surfactants PCC and LSA were initially evaluated but failed to yield homogeneous preconcentrates with compositions comparable to those of the other ZS. As a result, they were excluded from further investigations. Only stable preconcentrates were diluted 1:100 in HBS (pH 7.4) and either allowed to self-emulsify or were emulsified using a probe sonicator. The resulting NE were characterized for droplet size, PDI, and zeta potential. Ultimately, ten formulations were selected based on their composition (Table 1). Although all ZS are globally neutral, the surfactants bearing anionic moieties on the outer surface (CHS and LDA) exhibited a slightly negative zeta potential (approximately −10 mV) at physiological pH. This phenomenon is consistent with previous reports indicating that the presence of certain chemical moieties, such as sulfonates, on nanoparticle surfaces confers a negative zeta potential on them [20, 21]. Under gastric conditions (pH ≈ 1.2), the zeta potentials of some NE formulations changed markedly (Table 1). LDA-, DOX-, and MAP-based formulations exhibited a distinctly positive zeta potential. In line with its pKa value, CHS appears to be only negligibly protonated at pH 1.2 and therefore maintains a near-neutral surface charge. Interestingly, the surface charge seems to depend not only on the pKa of the protonatable group but also on the intramolecular distribution of charges. Although DOX and LDA have similar pKa values (4.17 and 3.62, respectively), DOX-based NE, with its strongly localized charge and lack of larger charge-shielding groups, displayed a substantially higher zeta potential. Moreover, despite the significant difference in pKa (1.88 and 3.62, respectively), MAP- and LDA-based NE exhibited comparable zeta potentials at pH 1.2. This effect is likely attributable to the quaternary ammonium group located on the exterior of MAP, whereas in LDA the quaternary ammonium group is shielded by the acetate residue at the NE droplet surface. Finally, preconcentrates contained an oil phase ranging from 10% to 50% (w/w), a surfactant between 30% and 50% (w/w), and a co-solvent between 20% and 40% (w/w) (Table 1). This approach balanced compatibility and minimized compositional variability, establishing a consistent foundation for evaluating the influence of surfactant type on NE properties, including stability, cellular uptake, and mucus diffusion. Nonetheless, the size measurements revealed already first differences in the ability of the surfactants to form NE. Size differences between LDA, CHS, and DOX were rather negligible, but the two MAP-based formulations exhibited significantly lower particle sizes than all other formulations. Notably, according to our HLB calculation (Table 1), MAP has the largest hydrophilic substructure of the investigated surfactants. The phosphorylcholine moiety leads to more pronounced interactions with the aqueous environment at the interface through hydrogen binding and ionic interactions stability [22]. Importantly, the phosphate- and quaternary ammonium-groups are able to interact with one another in neighboring surfactant molecules, therefore creating a tight network of ionic bonds on the surface of the oil phase which seemed to drastically reduce the size of the NE [23].

**Table 1:** Preconcentrates composition and corresponding NE characteristics: size and zeta potential are expressed as mean ± standard deviation (S.D.) of five different experiments

Samples	Oil Phase (% w/w)	Surfactant (% w/w)	Co-Solvent (% w/w)	Size (nm)	PDI	Zeta Potential at pH 7.4 (mV)	Zeta Potential at pH 1.2 (mV)
LDA1	Capmul^®^ MCM C8 : Captex^®^ 355 EP/NF – 50	Lauryl betaine − 30 HLB: 7.4	Transcutol^®^ HP – 20	202.3 ± 4.1	0.111 ± 0.022	-11.2 ± 1.1	25.7 ± 2.7
LDA2			Ethanol – 20	222.5 ± 1.3	0.210 ± 0.012	-7.3 ± 1.2	26.4 ± 1.1
CHS1	Capmul^®^ MCM C8 – 20	Cocamidopropyl hydroxysultaine − 50 HLB: 8.7	Transcutol^®^ HP – 30	192.9 ± 6.7	0.251 ± 0.022	-10.2 ± 1.2	-6.3 ± 2.0
CHS2			Ethanol – 30	200.8 ± 5.8	0.282 ± 0.012	-11.2 ± 2.1	-10.1 ± 0.7
CHS3	Capmul^®^ MCM C8 – 10		Transcutol^®^ HP – 40	161.2 ± 1.2	0.081 ± 0.009	-12.3 ± 0.7	-14.4 ± 1.8
CHS4	Capmul^®^ MCM C8 – 12.5		Transcutol^®^ HP – 37.5	170.1 ± 2.5	0.099 ± 0.030	-9.8 ± 2.2	-4.4 ± 1.2
DOX1	Capmul^®^ MCM C8 : Captex^®^ 355 EP/NF – 40	Lauryldimethylamine oxide − 40 HLB: 5.2	Transcutol^®^ HP – 20	197.3 ± 2.1	0.130 ± 0.033	-1.0 ± 0.1	53.9 ± 0.7
DOX2		Lauryldimethylamine oxide − 30 HLB: 5.2	Ethanol – 30	185.2 ± 2.2	0.151 ± 0.023	-0.8 ± 0.1	56.2 ± 2.3
MAP1	Capmul^®^ MCM C8 : Captex^®^ 355 EP/NF – 50	n-Dodecylphosphocholine − 30 HLB: 8.8	Transcutol^®^ HP – 20	69.8 ± 3.3	0.090 ± 0.021	-2.1 ± 0.3	23.9 ± 0.8
MAP2			Ethanol – 20	63.2 ± 1.7	0.161 ± 0.023	-1.4 ± 0.2	23.1 ± 1.4

### Stability study

**Fig. 2:**
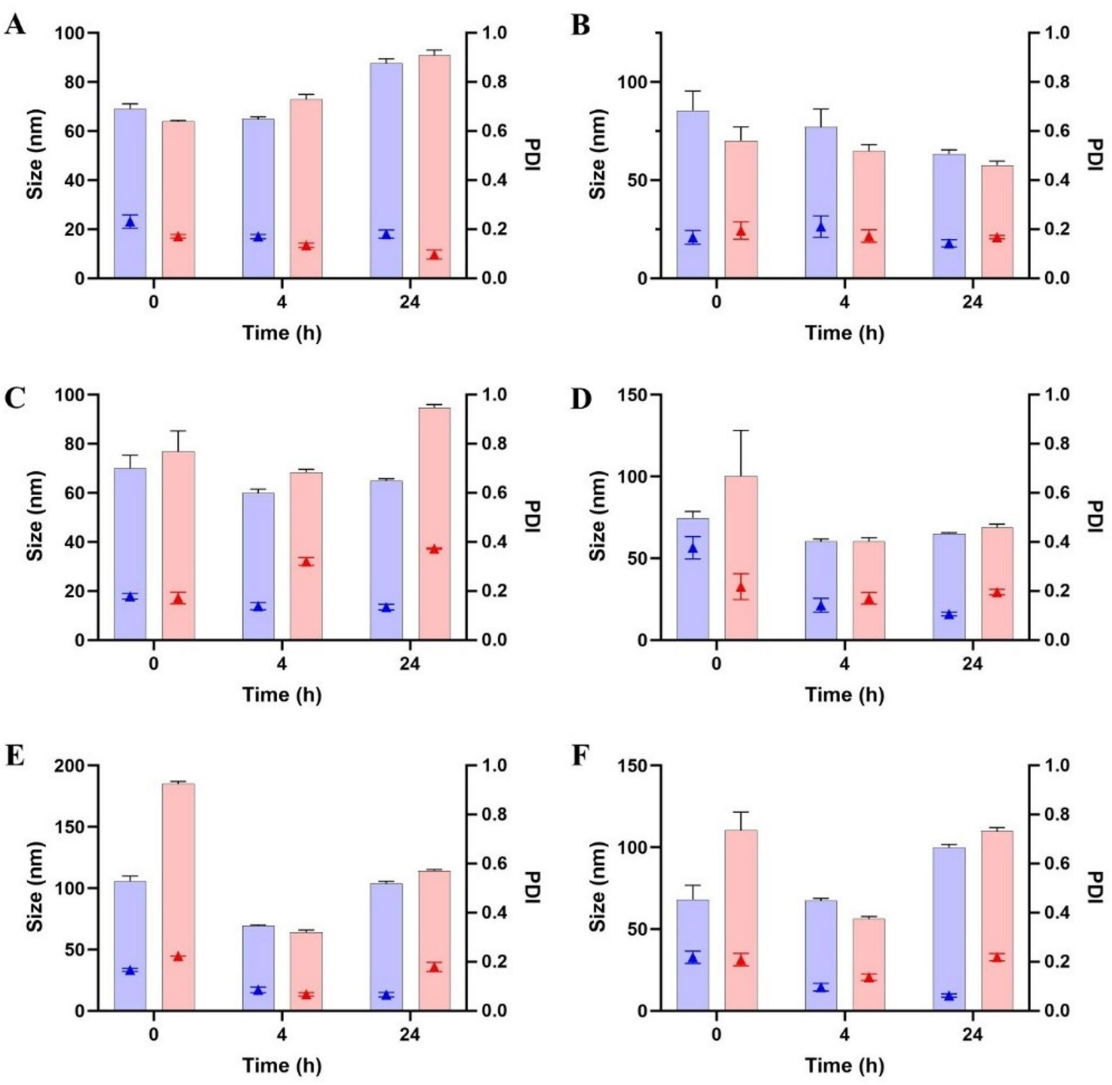
Size (filled bars) and PDI (triangle symbols) of 1% (V/V) MAP-based formulations in HBS (**A**), BSA solution (**B**), FaSSGF (**C**), FaSSGF + pepsin (**D**), FaSSIF (**E**), and mucus solution (**F**). Blue bars refer to NE MAP1 (TSC-containing) and red ones refer to MAP2 (EtOH-containing). Data are shown as mean ± SD (*n* = 5)

**Fig. 3:**
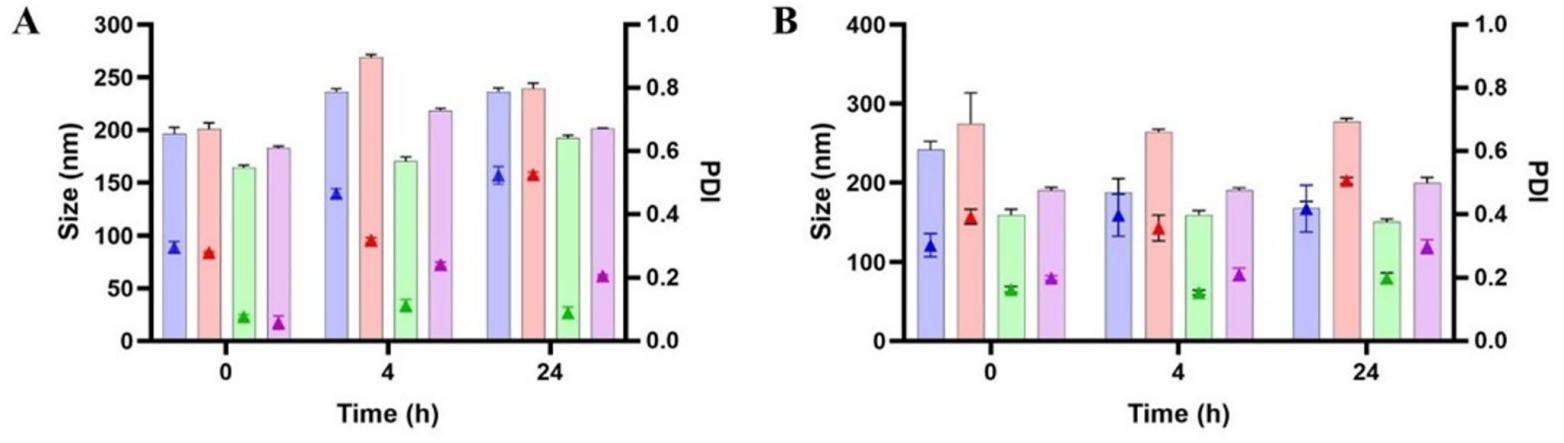
Size (filled bars) and PDI (triangle symbols) of 1% (V/V) CHS-based formulations in HBS (**A**) and BSA solution (**B**). Blue bars refer to CHS1, red bars refer to CHS2, green bars refer to CHS3 and purple bars refer to CHS4. Data are shown as mean ± SD (*n* = 5)

**Fig. 4:**
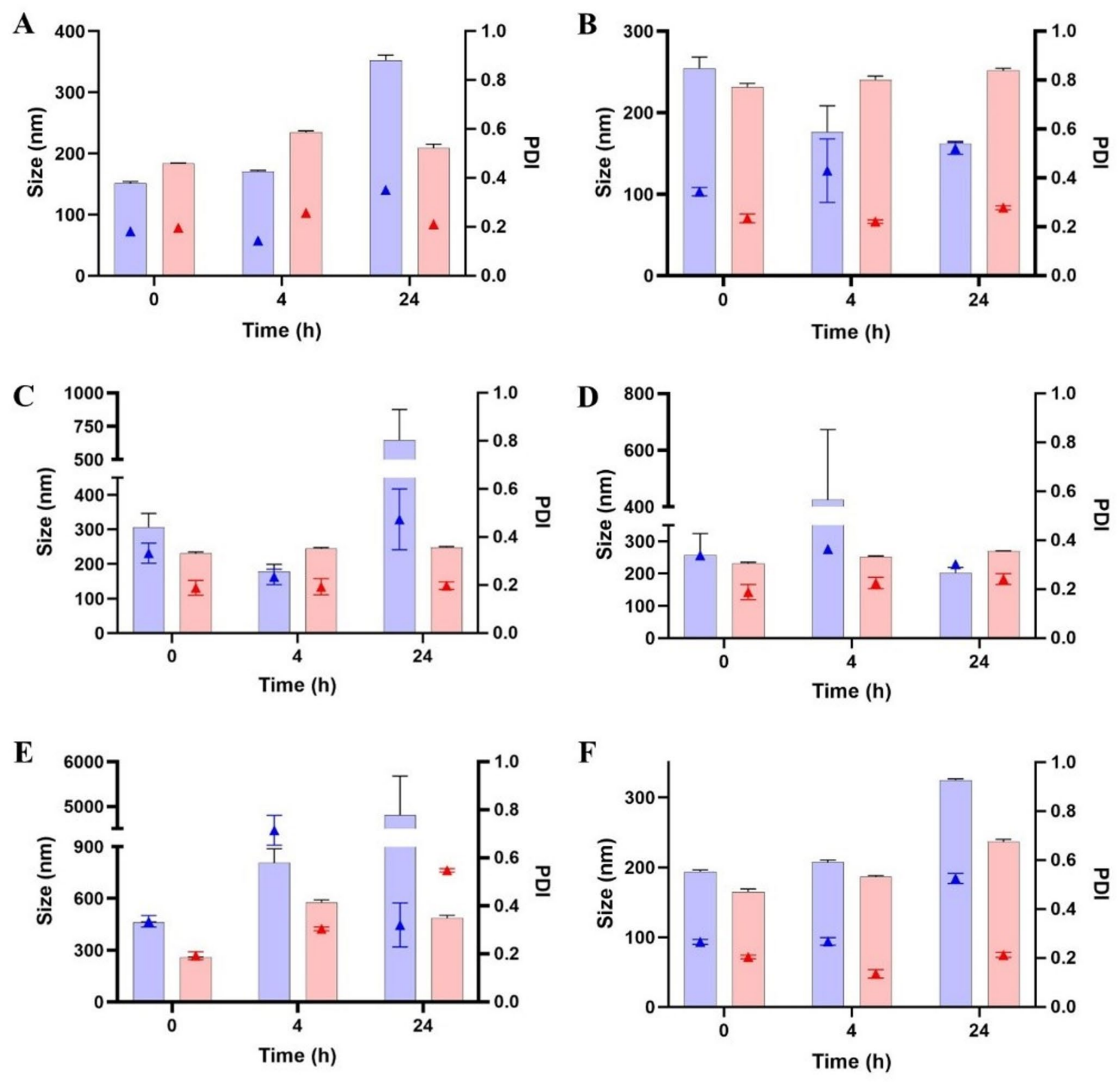
Size (filled bars) and PDI (triangle symbols) of 1% (V/V) DOX-based formulations in HBS (**A**), BSA solution (**B**), FaSSGF (**C**), FaSSGF + pepsin (**D**), FaSSIF (**E**), and mucus solution (**F**). Blue bars refer to NE DOX1 (TSC-containing) and red ones refer to DOX2 (EtOH-containing). Data are shown as mean ± SD (*n* = 5)

**Fig. 5:**
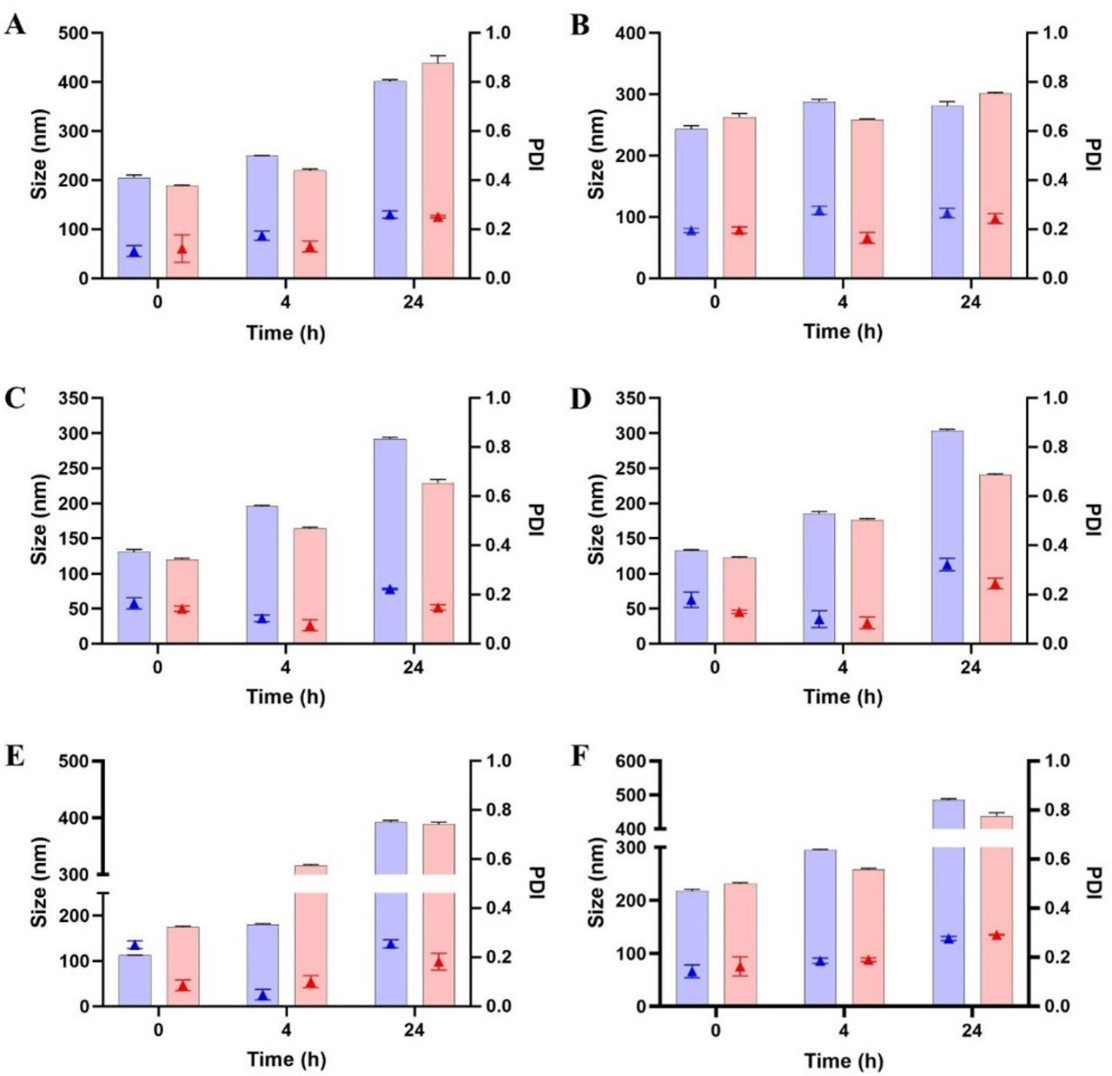
Size (filled bars) and PDI (triangle symbols) of 1% (V/V) LDA-based formulations in HBS (**A**), BSA solution (**B**), FaSSGF (**C**), FaSSGF + pepsin (**D**), FaSSIF (**E**), and mucus solution (**F**). Blue bars refer to NE LDA1 (TSC-containing) and red ones refer to LDA2 (EtOH-containing). Data are shown as mean ± SD (*n* = 5)

Substantial differences were observed depending on the surfactant structure and the co-solvent employed. In HBS (pH 7.4), MAP-based formulations exhibited good stability, with consistently low droplet sizes and PDI values over 24 h and no fluctuations (Fig. 2A). A clear distinction was observed between CHS-based SEDDS formulations (CHS3 and CHS4) and their non-self-emulsifying NE counterparts (CHS1 and CHS2). In HBS (pH 7.4), CHS3 and CHS4 exhibited good stability over 24 h, comparable to MAP-based formulations, with droplet sizes consistently below 250 nm and low PDI values (< 0.3) (Fig. 3A). Conversely, CHS1 and CHS2 showed a tendency to aggregate over time, but they were still considered stable enough to proceed with further investigations. Overall, the stabilizing role of CHS at neutral pH was demonstrated. In contrast, both DOX- and LDA-based formulations showed progressive increases in size and PDI over time (Figs. 4A and 5A). These formulations maintained stability over the first 4 h, with only slight increases in size and PDI. However, after 24 h, significant droplet size growth and PDI increases were observed, indicating droplet coalescence and reduced interfacial rigidity over time [20].

In protein-rich environments (BSA), MAP-based NE displayed good stability, showing only minimal changes in droplet size and PDI (Fig. 2B). The phosphorylcholine group has the ability to strongly bind water molecules on the surface, creating a hydration layer. This hydrophilic shield may have limited or prevented protein bridging and adsorption at the droplet surface, thereby maintaining interfacial stability [22]. CHS-based formulations exhibited similar behavior, showing overall moderate stability over 24 h, with only a minor increase in PDI for both self-emulsifying and mechanically emulsified NE formulations at the final time point, likely due to protein adsorption and bridging effects (Fig. 3B). Despite this, self-emulsifying formulations retained smaller droplet sizes, suggesting improved interfacial stabilization. BSA can promote interactions with the CHS-coated interface, leading to the displacement of interfacial surfactant molecules and subsequent destabilization. Conversely, for LDA- and DOX-based formulations, the droplet size was slightly higher compared to the stability test in HBS, suggesting moderate levels of protein adsorption or interaction with BSA at the interface (Figs. 4B and 5B). Despite this, they maintained relatively stable droplet size and PDI values over the 24-hour period.

In acidic conditions (FaSSGF and FaSSGF + pepsin), MAP-based formulations maintained moderate overall stability (Fig. 2C-D), likely due to the resistance of the phosphorylcholine group to protonation, thereby preserving its zwitterionic nature and preventing charge-induced aggregation [24]. In contrast, CHS-based formulations exhibited pronounced instability, with increases in droplet size and PDI up to the formation of visible aggregates, which made the samples immeasurable. Nanoparticles coated with sulfonate groups have been reported to exhibit a tendency to aggregate, especially in deionized water [25]. Regarding DOX-based NE (Fig. 4C), DOX2 (which contained EtOH) showed good stability, with consistent droplet size and PDI values over time. Conversely, in the case of the TSC-containing DOX1, pronounced instability was observed, suggesting reduced interfacial protection under acidic conditions. When pepsin was introduced (Fig. 4D), while DOX1 maintained the same trend, DOX2 remained stable, showing only a moderate increase in PDI, suggesting minor rearrangements at the droplet interface. Similarly, LDA-based NE (1 and 2) were only moderately stable in acidic environments, with the TSC-containing formulation showing slightly higher instability (Fig. 5C-D). However, the presence of pepsin exacerbated the destabilization, leading to greater PDI fluctuations after 24 h.

In FaSSIF, sodium taurocholate and lecithin introduced an additional destabilizing factor by competing with surfactant molecules at the droplet interface. This effect was evident across CHS-, DOX-, and LDA-based formulations, which displayed pronounced instability, with noticeable increases in droplet size and PDI over time (Figs. 4E and 5E). Nevertheless, MAP-based NE maintained structural integrity after an initial decrease in size for the EtOH-containing MAP2 (Fig. 2E). Fluctuations in droplet size and PDI were minimal compared to the other systems. The presence of bile salts and lecithin in FaSSIF can often lead to the competitive displacement of surfactant molecules, destabilizing the interface. However, the zwitterionic phosphorylcholine group in MAP exhibited an inert interfacial behavior, reducing interactions with sodium taurocholate and lecithin and thereby preserving NE stability. This characteristic further aligns MAP-based NE with other phospholipid-like systems, such as lecithin-based formulations, which are known for their biocompatibility and stability in intestinal conditions.

In mucus solution, MAP-based formulations again stood out, showing only minimal increases in droplet size and PDI (Fig. 2F), likely due to the phosphorylcholine group’s bioinert nature, which reduces interactions with mucin glycoproteins [26]. CHS-based NE displayed pronounced instability in mucus solution, which may be attributed to the unique surface characteristics of the droplets. The presence of sulfonate groups on the droplet surface in aqueous environments can lead to a dual behavior: a subset of nanodroplets forms small aggregates, while the majority remain stable [25]. Thus, the observed instability may be explained by this phenomenon, whereas the stable droplets, which show a slightly negative surface charge (zeta potential ∼ -10 mV), are still repelled and likely prevent extensive interactions with mucin glycoproteins. DOX-based formulations did not show pronounced interactions with mucin glycoproteins for at least 4 h, probably due to their overall electrical neutrality (Fig. 4F). In particular, DOX2 (the DOX-based NE with EtOH) displayed good stability, maintaining controlled droplet size and PDI values compared to DOX1, which showed higher variability due to the presence of TSC. In comparison, LDA-based formulations exhibited moderate fluctuations in droplet size and PDI (Fig. 5F). However, interactions with mucin appeared to be less disruptive than in FaSSIF, with no visible aggregation, which might be due to the presence of the carboxylate group, which tends to repel the negatively charged mucin glycoproteins.

Overall, the MAP-based formulations consistently exhibited superior stability, highlighting the unique contribution of the phosphorylcholine group in maintaining droplet integrity across diverse environments. CHS formulations performed better in self-emulsifying systems, benefiting from a lower oil-to-surfactant ratio, which likely enhanced interfacial stabilization. However, CHS formulations were found to be stable only in HBS and BSA-containing solutions. DOX- and LDA-based formulations showed variable stability, with EtOH-based systems consistently outperforming those containing TSC. These observations underscore the importance of both the surfactant’s molecular architecture and the choice of co-solvent in determining the performance and stability of NE across different biorelevant conditions.

### Mucus diffusion

**Fig. 6:**
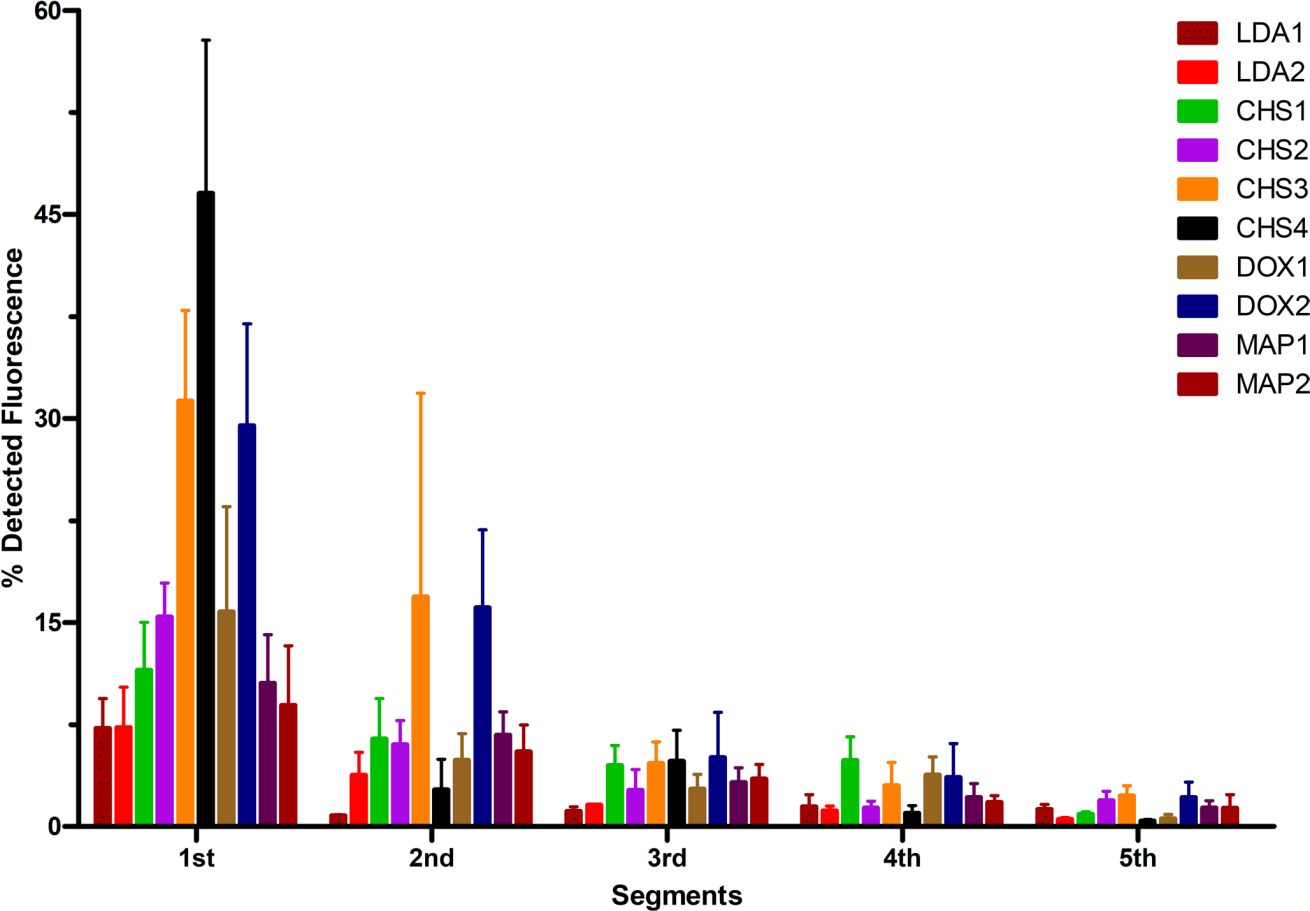
Percentage of Lumogen^®^ F Red 305 detected fluorescence evaluated in the first five segments. In each segment, the colored bars refer to the tested formulations. Results are expressed as mean ± S.D. (*n* = 3)

The mucus diffusion behavior of the tested NE was evaluated using a modified rotating cylinder method, with the fluorescence intensity of Lumogen^®^ F Red 305 serving as an indicator of mucus penetration depth (Fig. 6). Among the tested formulations, CHS3 and DOX2 exhibited the highest fluorescence intensities in the first and second segments, indicating superior diffusion capabilities into the mucus layers.

In stability studies, these formulations showed reduced physical stability in diluted mucus, an anomalous behavior that underscores the complexity of their interaction with mucosal environments. The higher diffusion observed for CHS3, despite its behavior in mucus solution, is likely attributable to the sultaine group. The increased mucus-permeating properties can be attributed to the outer sulfonate group in the zwitterionic headgroup and the overall negative surface charge of the formulation, which reduces electrostatic interactions with the negatively charged mucin network [27]. More specifically, the robust and large hydration shell as well as the stable negative charge of the outer sulfonate group likely prevent hydrophilic interactions between the mucus and the NE surface, leading to enhanced mucus penetration [28]. DOX2 also demonstrated higher diffusion than the other formulations; however, the underlying mechanism remains unclear, and further investigation is required. In contrast, MAP- and LDA-based formulations exhibited low diffusion. Despite the smaller particle size, which is generally favorable for mucus penetration, the behavior of MAP1 and MAP2 is consistent with that expected for a phosphocholine-like headgroup. Phosphocholines are known to interact with the mucins inside the intestinal mucus leading the NE to form aggregates with the mucus on its interface, and thus, hindering NE penetration into the mucus [29]. The diffusion characteristics of LDA-based formulations, however, are difficult to directly correlate with surfactant structure. Even though the negatively charged carboxy-group should sit outside on the NE surface, similar to CHS-based formulations, the diffusion properties were substantially lower. Compared to CHS, LDA’s carboxy-group has the ability to form strong hydrogen bonds with the mucus [30]. Furthermore, a thinner hydration shell could facilitate additional hydrophilic mucus interactions making it more likely for LDA-based formulations to be retained at the surface of the mucus [28]. As expected, fluorescence intensities decreased sharply in the third, fourth, and fifth segments for all formulations, reflecting the barrier properties of mucus. This trend aligns with the selective and layered architecture of the mucus network, which prevents deep penetration beyond the outermost layers. Overall, these findings highlight distinct differences in mucus diffusion behavior among the ZS, with CHS- and DOX-based formulations demonstrating enhanced penetration.

### Hemolysis assay

**Fig. 7:**
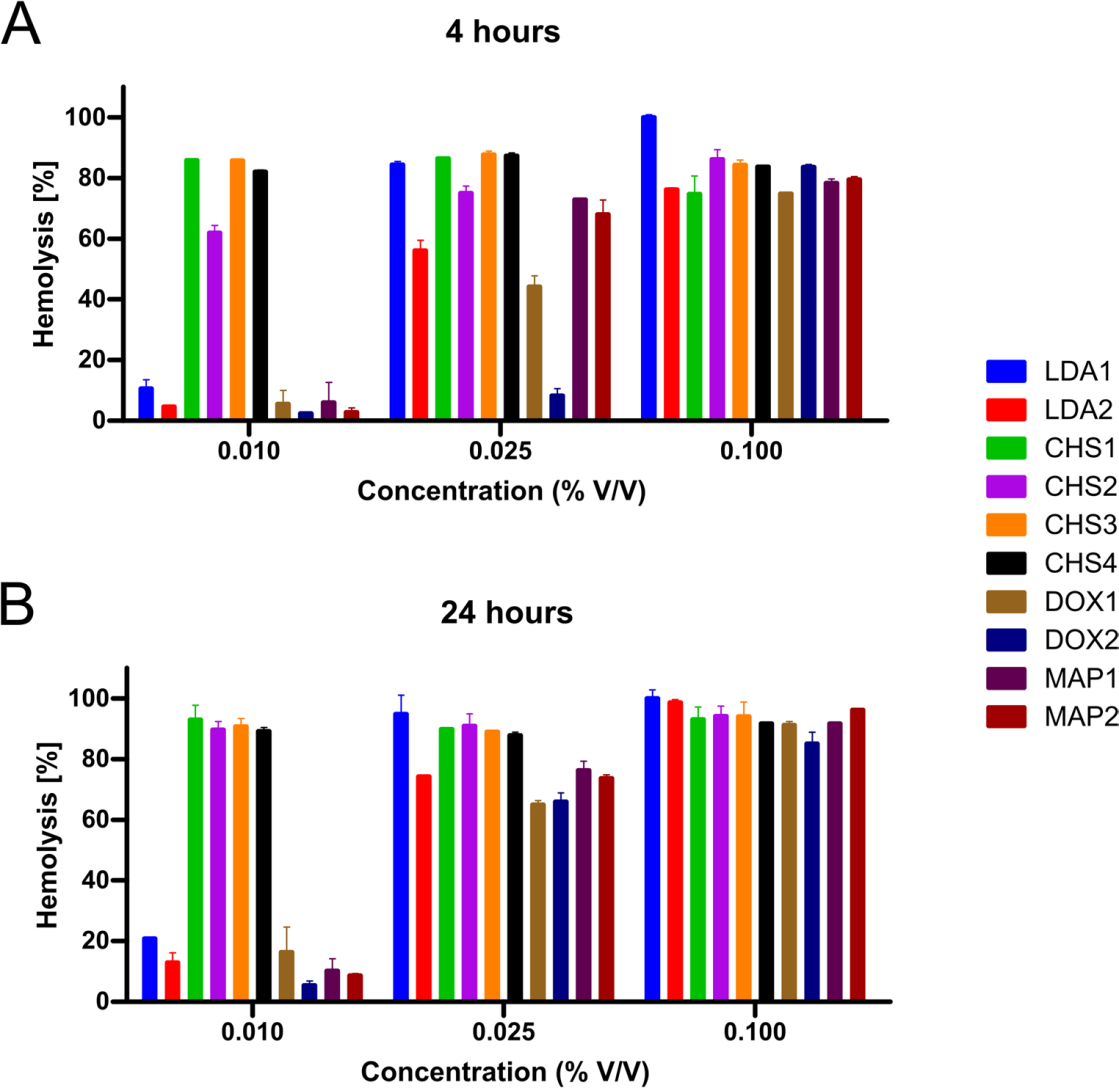
Hemolysis [%] of erythrocytes after 4 (**A**) and 24 h (**B**) of incubation with NE at the indicated concentrations. For each tested concentration, the colored bars refer to the tested formulations. Results are expressed as mean ± S.D. (*n* = 3)

The hemolysis assay provided critical insights into the biocompatibility and membrane interaction profiles of erythrocytes, i.e., potential to escape endosomes after endocytosis, for the tested formulations. A clear concentration- and time-dependent trend was observed, with hemolysis increasing proportionally across all formulations as both concentration and exposure time increased. After 4 h at the lowest concentration (0.01% v/v), CHS-based NE exhibited sustained hemolysis (∼ 80–100%), which persisted across all tested dilutions and time points (Fig. 7A). This pronounced hemolytic activity can be attributed to the high CHS concentration (50% w/w) and the potential of the quaternary ammonium group in the sultaine headgroup to strongly interact with erythrocyte membranes [31]. This behavior may imply enhanced interactions with cellular membranes and, consequently, a high cellular uptake potential. However, the toxicity of CHS-based formulations raises concerns regarding their suitability for systemic applications.

In contrast, DOX-based NE displayed a markedly lower hemolytic profile, particularly at lower concentrations (0.01% and 0.025% **v/v**). The reduced toxicity toward erythrocytes is likely due to the neutral polar headgroup of DOX, which minimizes membrane destabilization. DOX2 (30% w/w DOX) demonstrated significantly lower hemolysis than DOX1 (40% w/w DOX), underscoring that lower ZS concentrations lead to reduced hemolysis and suggesting an optimal balance between surfactant concentration and membrane interaction. MAP-based NE exhibited moderate-to-high hemolysis, which can be attributed to the phosphocholine-like headgroup of MAP. This structure facilitates strong interactions with erythrocyte phospholipid bilayers, particularly under prolonged exposure [32]. LDA-based NE showed intermediate hemolytic activity. The ammonium group in the betaine moiety of LDA may interact with erythrocyte membranes, accounting for its moderate hemolytic effect.

Notably, time dependence was evident across all formulations, with hemolysis consistently increasing after 24 h (Fig. 7B), reinforcing the importance of exposure duration in evaluating especially biocompatibility. These findings are consistent with literature reports indicating that higher surfactant concentrations often correlate with increased membrane toxicity, while the chemistry of the surfactant headgroup significantly modulates biocompatibility and delivery efficiency.

### Cell studies

#### Cytotoxicity

**Fig. 8:**
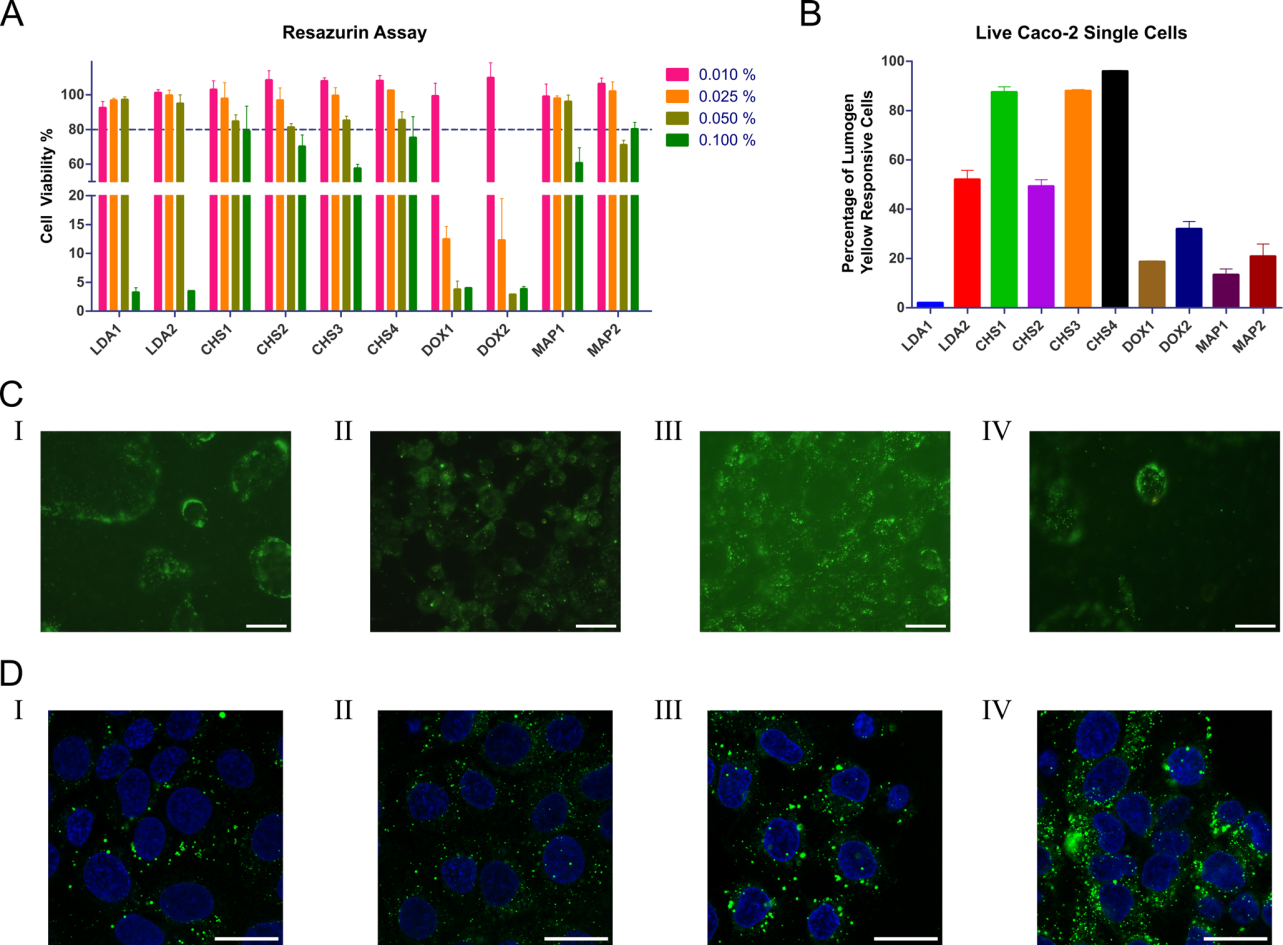
(**A**) Viability of Caco-2 cells after 4 h incubation. Each sample was tested at the following concentrations (% V/V): 0.01, 0.025, 0.05, and 0.1. Results are expressed as mean ± S.D. (*n* = 3). (**B**) Average amount of cells [%] displaying a fluorescence signal after incubation with the NE. Formulations were incubated on cells at a concentration of 0.01% (V/V) for 4 h. Results are expressed as mean ± S.D. (*n* = 4). (**C**) Fluorescence microscopy images of the formulations LDA2 (**I**), CHS4 (**II**), DOX2 **(III)**, and MAP2 (**IV**), scale bars (in white) are at 50 μm. (**D**) Confocal microscopy images of the formulations LDA2 (**I**), CHS4 (**II**), DOX2 **(III)**, and MAP2 (**IV**), scale bars (in white) are at 25 μm, nuclei are stained in blue, the formulations were labelled with Lumogen^®^ F Yellow 083 in green

The cytotoxicity of the formulations was evaluated using the resazurin assay on Caco-2 cells after 4 h of incubation (Fig. 8A). At the highest tested concentration (0.1% v/v), formulations containing MAP and CHS demonstrated moderate cytotoxicity, with cell viability generally remaining above 60%. In contrast, formulations containing DOX and LDA showed significant cytotoxicity, with cell viability dropping well below acceptable thresholds.

At intermediate concentrations (0.05% and 0.025% v/v), all formulations, except DOX-based NE (DOX1 and DOX2), maintained cell viability above 80%, classifying them as non-cytotoxic [33]. At the lowest concentration (0.01% v/v), all formulations, regardless of their ZS composition, were classified as non-cytotoxic, with cell viability consistently above 80%. This dilution likely minimizes membrane perturbation and mitigates any intracellular stress responses caused by the surfactants. Consequently, 0.01% (v/v) was selected as the optimal concentration for subsequent cellular uptake studies, ensuring both sufficient biocompatibility and functional surfactant activity.

This pattern diverges notably from the hemolysis assay results, where CHS-based formulations exhibited the highest hemolytic activity, while DOX-based formulations were among the least hemolytic. The observed cytotoxicity trends could be partly explained by the chemical structures of the ZS. DOX-based formulations demonstrated high cytotoxicity even at intermediate concentrations (0.05% and 0.025% v/v), a behavior that aligns with the reactive nature of amine oxide groups. Amine oxides are known to participate in redox reactions and can generate reactive oxygen species under certain conditions, leading to cellular oxidative stress and membrane damage. This structural reactivity likely contributes to the increased cytotoxicity observed in DOX-based formulations despite their relatively low hemolytic activity [34]. Conversely, MAP- and CHS-based formulations exhibited low cytotoxicity across all tested concentrations. MAP’s behavior is consistent with its phosphocholine-like headgroup. In several studies, lecithin, a phosphocholine-containing ZS, has been used as a ZS in lipid nanocarrier formulations. Phosphocholine structures are known to have high affinity for phospholipid bilayers, facilitating membrane integration without eliciting strong cytotoxic effects [8]. CHS-based formulations, despite their pronounced hemolytic activity, showed only moderate cytotoxicity on Caco-2 cells. This discrepancy may arise from differences in membrane composition between erythrocytes and Caco-2 cells [35, 36]. On the other hand, LDA-based formulations exhibited intermediate cytotoxicity profiles. The betaine headgroup of LDA and the negative zeta potential of the NE might mitigate interactions with cellular membranes. While this headgroup structure reduces overall membrane perturbation, it can still lead to membrane destabilization at high concentrations, contributing to moderate cytotoxicity.

#### Cellular uptake and fluorescence microscopy

NE cellular uptake was evaluated using flow cytometry and validated by fluorescence microscopy to assess the internalization of Lumogen^®^ F Yellow 083-loaded formulations in Caco-2 cells. Among the tested formulations, CHS-based NE demonstrated the highest uptake, with CHS4 achieving nearly 100% Lumogen^®^ F Yellow 083-responsive cells, indicating robust internalization (Fig. 8B). This behavior strongly correlates with the hemolysis assay results, where CHS-containing formulations exhibited pronounced membrane interaction properties due to their zwitterionic sultaine group, which facilitates strong electrostatic interactions with the membranes of erythrocytes, and therefore, allowing for endosomal escape. Fluorescence and confocal microscopy confirmed intracellular fluorescence, further substantiating effective cellular internalization (Fig. 8**CII and DII**).

DOX-based formulation DOX2 and LDA-based formulation LDA2 also displayed substantial uptake, as evidenced by significant percentages of Lumogen^®^ F Yellow 083-responsive cells. Fluorescence and confocal microscopy images of DOX2 revealed intracellular localization around the nuclei, supporting its uptake potential (Fig. 8**CIII and DIII**). On the other hand, despite flow cytometric data, fluorescence microscopy of LDA-based formulations highlighted more interactions between the NE and the cellular membrane than actual internalization of the formulation (Fig. 8**CI**). Subsequent confocal images confirmed only a low density of fluorescence signals inside the cells during optical cross-sectioning (Fig. 8**DI**).

In contrast, MAP-based formulations exhibited low cellular uptake. MAP2 predominantly interacted with the outer membrane, as shown in Fig. 8**CIV**, indicating minimal internalization. This behavior can be attributed to the phosphocholine-like headgroup of MAP, which stabilizes the outer lipid bilayer and limits translocation across the membrane. Surprisingly, optical cross-sectioning with confocal microscopy revealed a significant signal density inside the cells close to the nuclei (Fig. 8**DIV**). Interestingly, the signal is strongly centered and focussed onto single dot-like spots with low diffusion. It is therefore not unlikely that those spots were created through extensive cycling of the formulation after having been integrated into the cell membrane.

The study highlighted substantial differences in cellular uptake among the formulations depending on the employed ZS. These findings once again emphasize the distinct behavior of each surfactant, driven by its unique chemical structure and interaction with cellular membranes, thereby underlining the importance of tailoring ZS selection to the intended application.

Table 2 provides a systematic overview of the gathered results for all ten formulations.

**Table 2:** Systematic overview of results for the different formulations

Formulation	Surfactant	Stability	Mucus Diffusion	Hemolysis	Cytotoxicity	Cellular Uptake
LDA1	Lauryl betaine	Instable under intestinal and gastric conditions; time-dependent stability in (protein-rich) buffer	Low	Moderate	Moderate	Low
LDA2			Low	Moderate	Moderate	Low (membrane integration)
CHS1	Cocamidopropyl hydroxysultaine	Moderate stability in (protein-rich) buffer; instable under intestinal, gastric, and mucus conditions	Low	High	Low	High
CHS2			Moderate in the first segment	High	Low	High
CHS3		Good stability in (protein-rich) buffer; instable under intestinal, gastric, and mucus conditions	Superior	High	Low	High
CHS4			High in the first segment	High	Low	High
DOX1	Lauryldimethylamine oxide	Time-dependent stability in (protein-rich) buffer, moderately stable in mucus, instable under gastric and intestinal conditions	Moderate in the first segment	Moderate – Low	High	Low
DOX2		Good stability in (protein-rich) buffer and under gastric conditions, moderately stable in mucus, instable under intestinal conditions	Superior	Moderate – Low	High	Moderate – High
MAP1	n-Dodecylphosphocholine	Superior stability	Low	Moderate	Low	Low
MAP2			Low	Moderate	Low	Low (membrane integration)

## Conclusions

This study highlights the diverse performance of ZS in nanocarrier systems, revealing distinct advantages but also clear limitations that prevent any single surfactant from serving as an “ideal” candidate. MAP-based carriers showed superior stability and low to moderate cytotoxicity, favoring their use for membrane-targeting biologicals on mucosal surfaces. LDA formulations demonstrated low toxicity but lacked stability and mucus penetration. DOX carriers offered cellular uptake with low hemolysis, though cytotoxicity remained a drawback. CHS-based systems achieved the most balanced performance, with high uptake, effective mucus penetration, and benefits from self-emulsification, albeit limited by poor stability in bio-relevant media. Overall, surfactant structure critically determines stability, uptake, and barrier interactions. Studies as this one clearly show that zwitterionic surfaces can exhibit similar advantages as PEGylated ones without having to rely on the disadvantages of PEG. Future efforts should focus on identifying or combining ZS to integrate high stability, low toxicity, and efficient mucus penetration for advanced drug delivery applications.

## Supplementary Information

Below is the link to the electronic supplementary material.

Supplementary Material 1

## Data Availability

All data generated or analysed during this study are included in this published article and its supplementary information files.

## Abbreviations

ZS: Zwitterionic surfactant(s)
NE: Nanoemulsion(s)
PEG: Polyethylene glycol
CPT: Captex^®^ 355 EP/NF
CPM: Capmul^®^ MCM C8
TSC: Transcutol^®^ HP
BSA: Bovine serum albumin
EtOH: 96% (V/V) ethanol
MEM: Minimum essential medium eagle
HEPES: 4-(2-Hydroxyethyl)- 1-piperazinethansulfonic acid
PBS: Phosphate buffered saline
FBS: Fetal bovine serum
MAP: n-Dodecylphosphocholine (Mapcho^®^ 12)
LDA: Lauryl betaine
DOX: Lauryldimethylamine oxide
CHS: Cocamidopropyl hydroxysultaine
PCC: Palmitoyl-DL-carnitine chloride
LSA: Lauryl sultaine
HBS: HEPES buffer
PDI: Polydispersity index
FaSSIF: Fasted state simulated intestinal fluid
FaSSGF: Fasted state simulated gastric fluid
HLB: Hydrophile lipophile balance
