# Supplementary figures and images for: Exploring the impact of various zwitterionic surface modifications on the mucus diffusion and membrane permeability of lipid-based nanocarriers
Source: Drug Deliv Transl Res. 2025 Oct 9;16(6):1894–911. doi: 10.1007/s13346-025-01990-x (PMC13183736; doi:10.1007/s13346-025-01990-x)

**Supplementary Data**


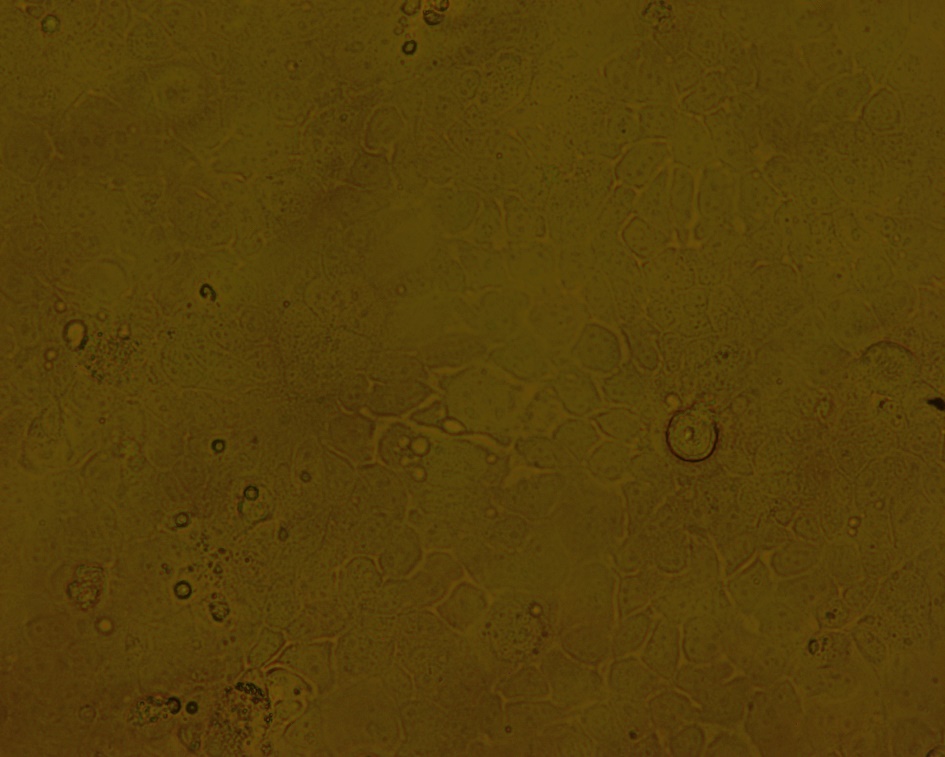


Figure S1: Bright-field Control Image (30X)


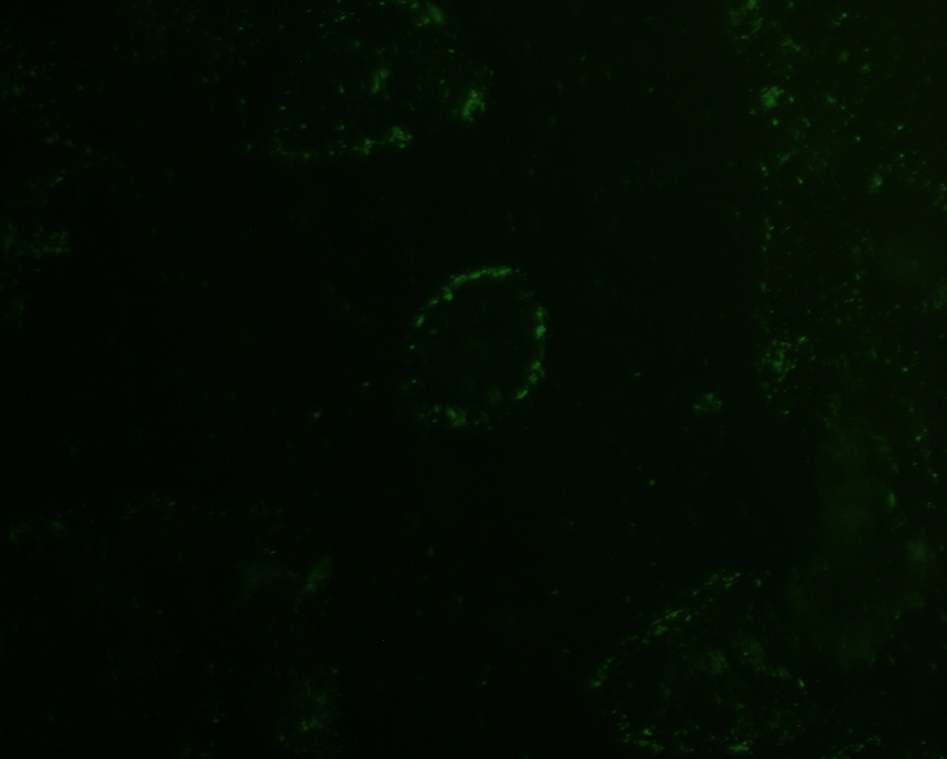


Figure S: LDA1 60X


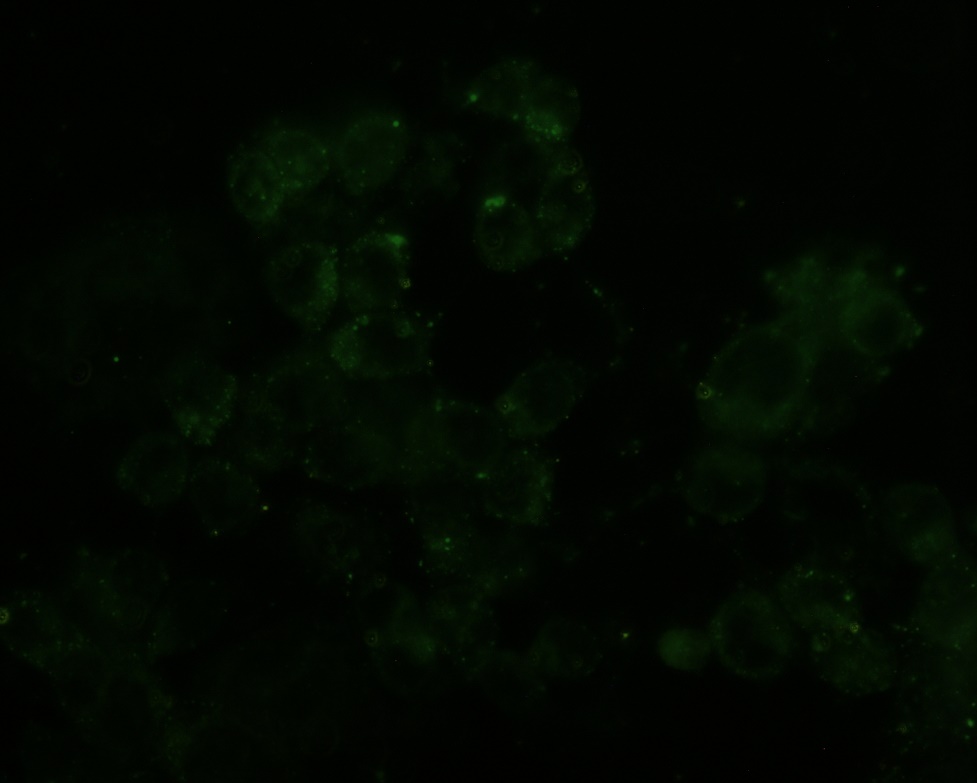


Figure S: CHS3 60X

Supplement: Supplementary file 1 — Supplementary Material 1 [file 13346_2025_1990_MOESM1_ESM.docx]
